# *De Novo* variants in the *KMT2A* (*MLL*) gene causing atypical Wiedemann-Steiner syndrome in two unrelated individuals identified by clinical exome sequencing

**DOI:** 10.1186/1471-2350-15-49

**Published:** 2014-05-01

**Authors:** Samuel P Strom, Reymundo Lozano, Hane Lee, Naghmeh Dorrani, John Mann, Patricia F O’Lague, Nicole Mans, Joshua L Deignan, Eric Vilain, Stanley F Nelson, Wayne W Grody, Fabiola Quintero-Rivera

**Affiliations:** 1 Clinical Genomics Center, David Geffen School of Medicine, University of California Los Angeles, Los Angeles, CA 90095, USA; 2 Departments of Pathology and Laboratory Medicine, David Geffen School of Medicine, University of California Los Angeles, 10833 Le Conte Avenue, Los Angeles, CA 90095, USA; 3 Department of Pediatrics, University of California Davis, Sacramento, CA, USA; 4 Department of Genetics, Kaiser Permanente, Fresno, CA, USA; 5 Department of Pediatrics, David Geffen School of Medicine, University of California Los Angeles, Los Angeles, CA 90095, USA; 6 Department of Human Genetics, David Geffen School of Medicine, University of California Los Angeles, Los Angeles, CA 90095, USA

**Keywords:** Wiedemann-Steiner syndrome, Clinical exome sequencing, *KMT2A*, Intellectual disability, Developmental delay

## Abstract

**Background:**

Wiedemann-Steiner Syndrome (WSS) is characterized by short stature, a variety of dysmorphic facial and skeletal features, characteristic hypertrichosis cubiti (excessive hair on the elbows), mild-to-moderate developmental delay and intellectual disability. [MIM#: 605130]. Here we report two unrelated children for whom clinical exome sequencing of parent-proband trios was performed at UCLA, resulting in a molecular diagnosis of WSS and atypical clinical presentation.

**Case presentation:**

For patient 1, clinical features at 9 years of age included developmental delay, craniofacial abnormalities, and multiple minor anomalies. Patient 2 presented at 1 year of age with developmental delay, microphthalmia, partial 3–4 left hand syndactyly, and craniofacial abnormalities. A *de novo* missense c.4342T>C variant and a *de novo* splice site c.4086+G>A variant were identified in the *KMT2A* gene in patients 1 and 2, respectively.

**Conclusions:**

Based on the clinical and molecular findings, both patients appear to have novel presentations of WSS. As the hallmark hypertrichosis cubiti was not initially appreciated in either case, this syndrome was not suspected during the clinical evaluation. This report expands the phenotypic spectrum of the clinical phenotypes and *KMT2A* variants associated with WSS.

## Background

Patients presenting with developmental delay and multiple dysmorphic features are a common diagnostic challenge in the genetics clinic. Over the past decade, many new genetic syndromes have been identified within this area. A significant number of these have been linked to genes involved in histone modification and chromatin remodeling. These include: Kabuki syndrome types 1 and 2 [MIM:147920 and 300867] [1,2], Kleefstra syndrome [MIM: 610253] [3], *KAT6B*-related disorders [MIM: 606170 and 603736] [4], Weaver syndrome [MIM: 277590] [5], *HDAC8*-related disorders [MIM:30882 and 309585] [6-8], and Wiedemann-Steiner syndrome [MIM: 605130] [9]. These along with Rubenstein-Taybi [MIM: 180849] [10] and Sotos Syndrome [MIM 117550] [11] make up a broad range of conditions cause by defects in chromatin remodeling genes. Similar to the loss of epigenetic control seen in Rett Syndrome [MIM: 312750], these disorders are thought to result from global changes in gene expression throughout development leading to abnormalities in multiple body systems. The majority of individuals with these disorders have impaired brain development leading to developmental delay and/or intellectual disability.

As these chromatin remodeling defect disorders are rare, with some having only a small number of cases reported, the complete phenotypic spectrum of many of them has not been well described. Thus while careful phenotyping remains critical for clinical diagnosis, it will often be insufficient to distinguish between related disorders. Genome-wide clinical tests such as SNP-based chromosomal microarray testing (SNP-CMA), clinical exome sequencing (CES), and clinical genome sequencing are incredibly powerful tools at identifying disease-causing variants in these genes: SNP CMA detection rate for ID ranges between 10-24% [12], while the diagnostic yield of exome sequencing, in patients with normal CMA results, ranges 10-40% [13].

Also of note is the strong pattern of *de novo* variants observed in many chromatin remodeling disorders [1,2,4,9,14]. Complete parent-proband trio sequencing is warranted in cases with developmental delay and dysmorphic features, as it has the power to directly identify *de novo* variants. In addition to expediting the process of identifying *de novo* variants in the known chromatin remodeling genes, there are many histone modification genes which have not been associated with human disease [15]. With complete trio clinical exome sequencing, it is possible to identify candidate novel disease gene associations using clinical information and predictive molecular tools.

The two patients presented in this report were seen at different medical institutions and by separate medical teams. Based on the reported clinical findings, there was no *a priori* expectation from within the clinical laboratory that these two individuals were connected in any way. Clinical exome sequencing was performed on full trios in both cases using clinically validated protocols (Additional file 1), detecting unique *de novo* likely pathogenic variants in the *KMT2A* (*MLL*) gene in each patient.

Fusions between the *KMT2A* gene with a variety of other genes are commonly observed in leukemic cells [16,17], giving the gene its original name: “myeloid/lymphoid or mixed lineage leukemia gene” or *MLL. KMT2A* is widely expressed, detectable in most human tissues [18]. It contains 36 exons and has three known mRNA isoforms (NM_001197104.1, NM_005933.3, and NM_024891.2). It is a homologue of the *d. melanogaster* gene trithorax. Mice heterozygous for a knockout mutation of the homologous *Ktm2a* gene exhibit retarded growth, skeletal and hematopoietic abnormalities [19,20]. The *KMT2A* gene product KMT2A contains several functional domains. One domain is a SET domain which acts as a histone H3 lysine 4-specific methyltransferase, thus regulating a variety of developmental genes including those in the HOX family [21].

Wiedemann-Steiner Syndrome has been described as a clinical entity defined by the presence of hypertrichosis cubiti (hairy elbows) and variable presentation of additional features such as facial dysmorphism, short stature, intellectual disability, and developmental delay [22-25]. In an exome sequencing study of WSS, *de novo* DNA variants in the *KMT2A* gene were identified in five out of six patients, strongly implicating this gene as the major disease gene for WSS [9].

## Case presentation

### Patient 1: 9 year-old female of Mexican ancestry

#### Pre/perinatal history

Prenatal course was normal with vaginal delivery. The birth weight (3.4 kg, 50–75 centile) and length (49.5 cm, 50-75 centile) were both normal. Head circumference was not available.

#### Review of systems

Poor muscle tone was noted at birth, and by 7 months of age significant general hypotonia and muscle weakness were apparent. At 20 months her development was significantly delayed, both for motor skills (due to continued hypotonia) and speech production. Poor feeding was noted at this time. Growth parameters at this age were markedly low, being below the 3^rd^ centile in height, weight, and head circumference. Receptive language appeared normal. At 4.5 years of age, premature dental eruption of adult teeth was noted. Ophthalmic exams were normal except for correctable astigmatism. Upon intellectual ability testing at approximately age 5, she had very poor scores for “spatial ability”, “general conceptual ability” and “special nonverbal composite”. She scored as average for “nonverbal reasoning ability” and below average for “verbal ability”. These scores represent an estimated IQ of approximately 65–75.

#### Physical exam

At approximately 10 years of age: Height is 165 cm (>95 centile); weight is 50 kg (95 centile).

#### Dysmorphic features

**Figure 1:**
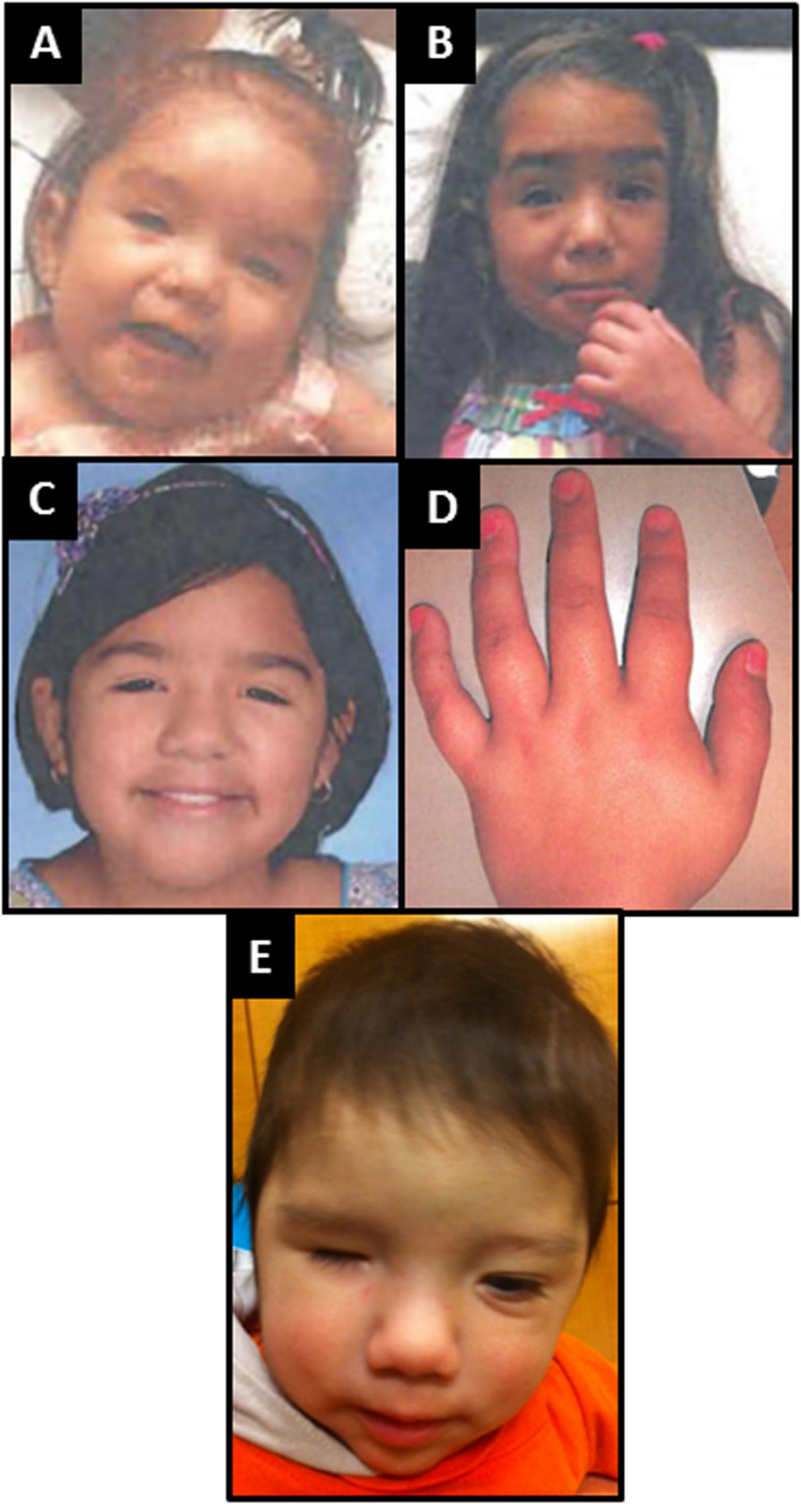
Features of patient 1 (A-D) and patient 2 (E).

Hypertelorism, bulbous nose, clinodactyly, downslanting and short palpebral fissures, a wide and depressed nasal bridge, thick eyebrows and hair, long thick eyelashes, synophris, thin lips, hypertelorism (Figure 1A-D, Table 1).

**Table 1:** **Comparison of clinical features of patients with *MLL-*** **related WSS**[9]**with patient 1 and patient 2**

**System**	**Phenotype**	**WSS n = 5**	**Patient 1**	**Patient 2**
**Growth**	Intrauterine growth restriction	3	-	/c
	Postnatal growth restriction	5	+ a	+d
**Nervous**	Developmental Delay	5	+	+
	Hypotonia	n.a.	+	+
	Mild Intellectual disability	5	+b	n.a.
**Craniofacial**	Strabismus	n.a.	+	-
	Microcephaly	2	- a	-d
	Wide anterior fontanelle	0	-	+
	Wide nasal bridge/broad nasal tip	5	+	+
	Downslanting/small palpebral fissures	5	+	+
	Hypertelorism	?	+	+
	Right microphthalmia	0	-	+
	Micrognathia	0	-	+
	Upper vermillion border thin	3	+	n.a.
**Skeletal**	Tapering fingers	3	+	+
	Broad 1^st^ digit	2	-	+
	Sacral dimple	4	+	+
	Rib anomalies (X-ray)	3	n.a.	n.a.
	2-3 Toe Syndactyly	0	-	+
	5^th^ finger clinodactyly	n.a.	+	n.a.
**Integumentary**	Hypertrichosis	5	/	-
	Hypertrichosis cubiti	5	/	-
	Long/thick eyelashes	5	+	+
	Thick eyebrows	5	+e	+
	Thick hair	n.a.	+	+

Legend: + Present; − Absent/questionably present; n.a. not ascertained. a. height (ht.), weight (wt.), head circumference (OFC) <3rd centile; b. IQ 65–75; c. birth wt. & OFC normal, ht. < 5^th^ centile. d. wt. and ht. (<3rd centile), OFC (<5th centile). e. synophris.

#### Previous testing

The following tests were all negative/normal: array comparative genomic hybridization, Fragile X Syndrome (*FMR1* triplet repeat expansion); Rett Syndrome (*MECP2* sequencing); Coffin-Lowry Syndrome (*RPS6KA3* sequencing); myotonic dystrophy (*DMPK* sequencing); Prader-Willi Syndrome (by methylation) 3-hydroxyisobutric aciduria (valium load testing).

#### Family history

Family history is unremarkable, with one unaffected sibling who does not share any of the clinical features noted here. Consanguinity is denied.

#### Therapy and other interventions

She is enrolled in special education classes and receives regular speech and occupational therapy. Carnitine supplementation for hypotonia was given with limited response. She had multiple procedures to remove teeth, and has suffered multiple urinary tract infections.

### Patient 2: 1 year-old male of Caucasian (maternal) and Mexican (paternal) ancestry

#### Pre/perinatal history

He was born by Cesarean section at 38 weeks of gestation, following an uncomplicated pregnancy, to a 21 year old mother and a 26 year-old father. The birth weight (3.02 kg, 25-50 centile) and head circumference (34.9 cm, 50-75 centile) were both normal, but the birth length of 44.4 cm was below the fifth centile. Dysmorphic features and microphthalmia of the right eye were noted at birth. An ocular prosthetic was placed at ~3 months of age.

#### Review of systems

He has a history of developmental delay beginning at 4 months of age when failed to achieve milestones (he did not roll over). At this time, vocalization was normal and object tracking was mildly impaired. He had a weak grip, significant central hypotonia, decreased muscle bulk, and head lagging. At the time of exam (1 year) he uses only one, non-specific word (‘dada’). He cannot sit up independently or crawl, but can “scoot”.

#### Physical exam

Weight 6.24 kg (<3^rd^ centile); length 67 cm (<3^rd^ centile); head circumference 44.1 cm (5^th^ centile); anterior fontanelle not fused (1.5 cm × 1.5 cm).

#### Dysmorphic features

Wight microphthalmia, micrognathia, wide nasal bridge, thick hair, low anterior hairline, two posterior hair whorls, long and prominent eyelashes, sacral hypertrichosis, small palpebral fissures which are down-slanted with telecanthus, tapered fingers, 3–4 partial left-hand syndactyly, mild pectus excavatum, and small feet and hands (Figure 1E, Table 1).

#### Previous testing

Normal karyotype (46, XY), SNP-CMA. Normal spinal canal ultrasound at 4 months of age, ruling out neural tube defects, and an abdominal ultrasound which ruled out gross malformations of the liver, gallbladder, pancreas, spleen, and kidneys. No brain MRI has been performed.

#### Family history

The family history is unremarkable except for a paternal half-brother who was born with a unilateral dysplastic kidney. Two other paternal half-siblings and one full sibling are healthy, as are the parents. Consanguinity is denied.

#### Therapy and other interventions

He receives weekly physical and occupational therapies (started at 5 months). Substantial gains have been made in the domains of motor functioning and expressive language.

##### Molecular testing

Exome Sequencing was performed in the UCLA Molecular Diagnostics Laboratories using clinically validated protocols. The proband and both parents were sequenced in each case (trio analysis). All genes harboring *de novo*, homozygous or compound heterozygous variants with allele frequencies <1% in the general population [26] were evaluated by a Genomics Data Board consisting of physicians, pathologists, clinical geneticists, laboratory directors, genetic counselors, and informatics specialists. See Additional file 1 for a detailed description of the bioinformatic methods used for exome sequencing analysis. Variant Annotator X (VAX) was used for rich annotation of DNA variants as previously described [27].

Criteria for high confidence for *de novo* variants was: quality score >=Q500 in each individual [28]; variant observed in <2 reads in any individual parent; variant not observed in the general population. Clinically significant variants were confirmed using PCR amplification and Sanger sequencing of the proband and both parents (Additional file 2).

### Genomic structure

Due to the high proportion of variants being inherited from one or the other parent (>99.9%), non-paternity was excluded in both cases. No homozygous blocks of >5 Mb were identified in either patient, indicating a very low probability of significant autozygosity. No apparent homozygous exon deletions were identified within the primary gene lists in either patient.

### Exome sequencing results

#### Patient 1

A primary gene list of 1,274 genes (Additional file 3) was generated by searching Human Gene Mutation Database Professional Version 2012.4 (HGMD), Online Mendelian Inheritance in Man (OMIM, searched February, 2013), and GeneTests.org for the following clinically relevant keywords: *developmental delay, (mild) mental retardation, intellectual disability, hypotonia, (probable) myopathy, ptosis, dysmorphic features, craniofacial abnormalities, hypertelorism, bulbous nose, clinodactyly, tapering fingers, downslanting palpebral fissures, wide nasal bridge, astigmatism, early tooth eruption, premature adult teeth.*

A total of 14,395,023,189 bases of DNA sequence were generated for Patient 1, resulting in an average read depth (“coverage”) of 157× across RefSeq coding positions, with 95% of all targeted positions covered by > =10 independent reads (Additional file 4). A total of 22,275 variants were identified within exomic loci (21,212 single nucleotide variants and 1,063 insertion/deletion variants) compared the human genome reference (hg19/NCBI Build 37).

**Figure 2:**
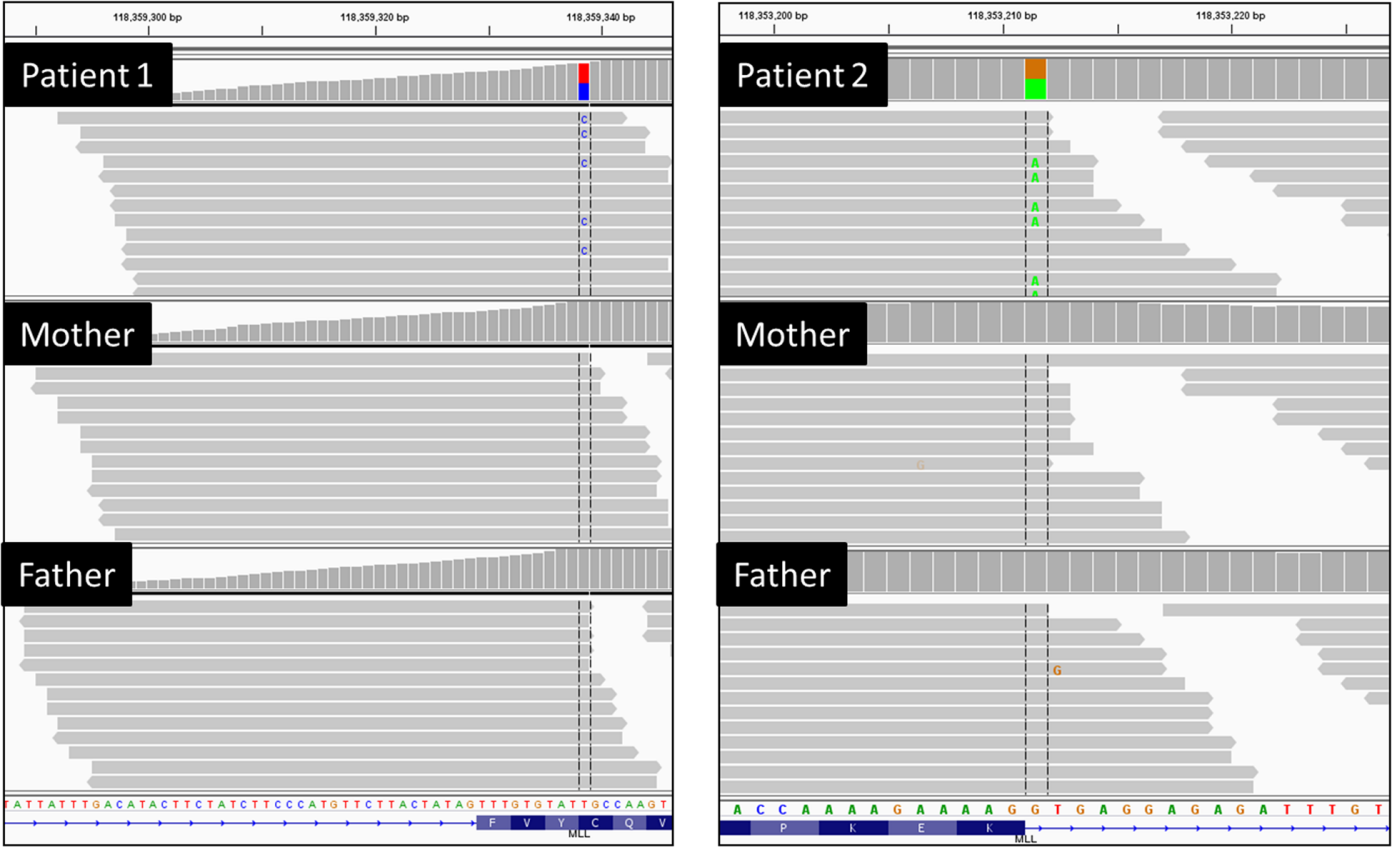
**View of aligned sequence reads spanning the *KMT2A *** **variants in both patients and their parents using the Integrated Genomics Viewer**[39]**.**

One *de novo* variant was identified within the primary gene list: a heterozygous c.4342T>C (p.Cys1448Arg) missense variant in the *KMT2A/MLL* gene (NM_001197104.1). Alignment view of this variant in the Integrative Genomics Viewer (IGV) [29] can be seen in Figure 2. *In silico* prediction was performed using functional prediction algorithms SIFT (0.00: “Affected Protein Function”) and PolyPhen2 (0.995: “probably damaging”). With over 200× coverage in the all three members of the trio, this variant is of high quality and coverage see (Additional file 4).

Other variants of uncertain clinical significance, likely benign, were identified in *VPS13B.* Detailed variant information for the above mentioned variants can be found in Additional file 5.

#### Patient 2

A primary gene list of 1,553 genes (Additional file 3). The following clinical keywords: *microphthalmia, poor growth, growth retardation, micrognathia, hypotonia, developmental delay, wide fontanelle, dysmorphic, turned-out hands, syndactyly, pectus excavatum*.

A total of 10,744,929,539 bases of DNA sequence were generated for Patient 2, resulting in an average read depth (“coverage”) of 127× across RefSeq coding positions, with 94% of all targeted positions covered by > =10 independent reads (Additional file 4: Materials 3b and 4). A total of 22,751 variants were identified within exomic loci (21,498 single nucleotide variants and 1,253 insertion/deletion variants) compared the human genome reference (hg19/NCBI Build 37).

One *de novo* variant was observed within the primary gene list: a heterozygous c.4086+1G>A variant in the *KMT2A/MLL* gene (NM_001197104.1). Alignment view of this variant in the Integrative Genomics Viewer (IGV) [29] can be seen in Figure 2. With over 150× coverage in the all three members of the trio, this variant is of high quality and coverage (Additional file 4). As this variant occurs at the first nucleotide of intron 8 of the gene, it is predicted to result in a loss of function allele due to the abolition of the canonical splice donor site. However, the sequence of the mature mRNA produced by this allele cannot be predicted from sequence alone.

Other heterozygous variants of uncertain clinical significance in *GFI1B, PCDH15* and *MED13* were identified. Detailed variant information for the above mentioned variants can be found in Additional file 6. No other variants were identified in patient 2 which were consistent with an autosomal or X-linked recessive or *de novo* dominant mode of inheritance. No rare variants in genes associated with microphthalmia were found.

## Conclusions

A subset of WSS is caused by heterozygous *de novo* variants in the *KMT2A* (*MLL*) gene [9]. This subset is characterized by mild to moderate developmental delay, dysmorphic facial features (including: long eyelashes, thick or arched eyebrows, downslanting palperbral fissures, broad nasal bridge, and Cupid’s bow abnormality of the upper lip), and hypertrichosis cubiti (excessive hair on the elbows). A “slim and muscular build” was noted in 3/5 initial *KMT2A*-related WSS cases*.* Other features observed in some WSS patients include high narrow palate, tapering fingers, 5^th^ finger clinodactyly and hypotonia. The clinical spectrum of features associated with WSS is wide and may continue to expand as additional patients such as these are identified.

Exome sequencing results for these trios are suggestive of a molecular diagnosis of Wiedemann-Steiner Syndrome (WSS) in both patients. Our patients shares several of the features of *KMT2A*-associated WSS, including postnatal growth retardation, developmental delay, wide nasal bridge, broad/bulbous nasal tip, and downslanted palpebral fissures [9,30]. They do not however have clear hypertrichosis cubiti, the clinical feature most readily associated - but not pathognomonic - with WSS. In them the excess of hair is manifested by thick eyebrows and hair, long thick eyelashes, and the sacral hypertrichosis observed in patient 2 (Table 1). In one of our patients and one previously reported individual with WSS [9], there is history of recurrent infections, though it remains unclear whether their immune dysfunction is related to *KMT2A* mutation. Patient 2 has several clinical features not previously observed in individuals with WSS, including: unilateral microphthalmia, micrognathia, 3–4 finger syndactyly, and premature eruption of adult teeth.

The *de novo* variant identified in the *KMT2A* gene in patient 1 is a missense c.4342T>C variant. To date, all *KMT2A* variants reported in WSS patients are premature truncation variants, suggesting haploinsufficiency as the disease mechanism. As the c.4342T>C variant does not result in protein termination, the effect of this variant on KMT2A protein abundance and/or activity cannot be confidently predicted. However, this missense variant is located within a PDH homeodomain zinc finger domain, a domain thought to coordinate protein-protein interactions involved in transcriptional activation [31]. The web-based tool Human Splicing Finder v2.4.1 [32] was unable to provide a meaningful prediction as to whether this variant impacts splicing.

Given that typical human exomes carry between zero and five high confidence *de novo* coding variants [9,13,14,33-39] and the inclusive approach to generating the primary gene list (over 1,000 genes included in each case), the identification of a previously unreported *de novo* missense variant in the *KMT2A* gene in a single case is not by itself a significant finding. However, combined with the phenotypic overlap between individuals with *de novo* variants in *KMT2A* with WSS and these two unrelated patients, these findings strongly implicate a causal relationship between the observed variants and the clinical presentation of these individuals. Functional analysis or identification of other patients with the same variants and similar phenotypes would provide additional support.

This report highlights the value of full trio clinical exome sequencing for individuals with multiple congenital anomalies and developmental delay whose features are not consistent with one particular syndrome, supporting the model of medical genetics practice recently suggested by Shashi and colleagues [39]. Without parental sequences, the variants in *KMT2A* would not have been singled out from among many similar heterozygous candidate variants identified within the primary gene list. Thus full trio exome sequencing greatly improved the interpretability of the test in these patients.

Financial considerations are also an important factor in molecular testing. Full trio clinical exome sequencing is comparable in cost to gene panel testing [40] and, if pursued as a second-line test after clinical microarray analysis (SNP-CMA), is likely a far more efficient use of resources than iterative single gene testing in cases with developmental delay and dysmorphic features.

### Consent

For both patients, a parent or legal guardian consented to the following statement: “We [the UCLA Clinical Genomics Center] will use your results to improve Clinical Exome Sequencing by comparing your data to others”. Additional written consent was acquired for both patients for the use of their photographs for research publication.

### Ethics statement

As the genetic testing data were obtaining using a clinical test and appropriate written consent for testing was obtained, this report is exempt from ethics approval for medical research of human subjects. All authors have received training and are compliant with the Health Information Portability and Accountability Act of 1996 (HIPAA).

## Competing interests

SPS, JLD, KD, HL, FQ-R, and WWG work for a fee for service laboratory providing diagnostic testing. The remaining authors declare that they have no competing interests.

## Authors’ contributions

SPS performed analysis and prepared the manuscript. RL and NM provided phenotype information and photographs for patient #2. HL performed analysis and interpretation of molecular testing. ND served as liaison between sites and contributed to the description of phenotypes for both cases. JM provided phenotype information and photographs for patient #1. PFO provided genetic counseling and phenotype information for patient #1. JLD, EV, SFN, and WWG participated in the study design and provided clinical laboratory testing for both cases. FQ-R conceived of the study, and participated in its design and coordination, and the Genomic data board. All authors read and approved the final manuscript.

## Author’s information

SPS is the submitting author.

## Pre-publication history

The pre-publication history for this paper can be accessed here:

http://www.biomedcentral.com/1471-2350/15/49/prepub

## Supplementary Material

Additional file 1 Bioinformatic Methods used for Data Analysis of Next Generation Sequencing Results. Click here for file

Additional file 2 **Sanger sequencing traces confirming *de novo* variants in the *KMT2A* gene.** Click here for file

Additional file 3 Primary gene list for patients 1 and 2. Click here for file

Additional file 4 Sequencing statistics and variant counts for all individuals. Click here for file

Additional file 5 Variant table for patient 1. Click here for file

Additional file 6 Variant table for patient 2. Click here for file
