# Supplementary material for: De Novo variants in the KMT2A (MLL) gene causing atypical Wiedemann-Steiner syndrome in two unrelated individuals identified by clinical exome sequencing
Source: BMC Med Genet. 2014 May 1;15:49. doi: 10.1186/1471-2350-15-49 (PMC4072606; doi:10.1186/1471-2350-15-49)
Supplement: Additional file 1 — Bioinformatic Methods used for Data Analysis of Next Generation Sequencing Results. [file 1471-2350-15-49-S1.doc]

**Bioinformatic Methods used for Data Analysis of Next Generation Sequencing Results**

The sequence reads were aligned to the Human reference genome, human_g1k_v37.fasta (downloaded from The Genome Analysis Toolkit (GATK) resource bundle in July, 2011) using Novoalign V2.07.15b from Novocraft with the adaptor stripping (-a) and hard clipping (-H) options on. SAMtools version 0.1.16 was used to sort the aligned BAM files and potential PCR duplicates were marked (rmdup) using Picard-tools-1.42. On average, 90% of the reads were uniquely aligned to the reference genome. Local realignment for each sample was performed using the GATK v1.1 ‘IndelRealigner’ tool and base qualities were recalibrated using the GATK v1.1 ‘TableRecalibration’ tool following GATK’s recommendation (Best Practice Variant Detection with the GATK v2). Variants were called using the GATK ‘Unified Genotyper’ tool using minimum 16 samples. The dbSNP132 file downloaded from the GATK resource bundle was used for known site resource. Only the variants found within the RefSeq protein coding regions +/-2bp were reported by using the –L option. Single nucleotide variants were recalibrated using GATK VariantRecalibrator with hapmap_3.3.b37.sites and 1000G_omni2.5.b37.sites for training resources and small INDELs were filtered using VariantFiltration.

The analytical performance characteristics of the UCLA Clinical Exome Sequencing have been evaluated according to the standards of the UCLA Molecular Diagnostics Laboratories. Accuracy has been evaluated at three levels: 1) single base pair level (by method comparison using Sanger sequencing), 2) across exome (by method comparison using Illumina HumanOmniExpress BeadChip) and 3) base-calling (as reported for each sequencing run in the metrics of a Q-score). The mean accuracy at single base pair was 100% with standard deviation (SD) = 0.01%. The accuracy of base-calling exceeded the manufacturer’s recommendations. Precision was measured by reproducibility and repeatability. Reproducibility was evaluated on three independent samples, with imprecision consistently less than 0.1% for SNVs and less than 3% for INDELs. Repeatability of the assay and stability of the reagents were evaluated for two independent samples over a three week period, and imprecision was consistently less than 0.1% for SNVs and less than 3% for INDELs. The PhiX error rate measured for each lane by HiSeq2000 or HiSeq2500 was consistently less than 1% and various metrics evaluated for both SNVs and INDELs were consistent across all libraries used in the validation study. These metrics are used as guidelines for internal quality control.
