# Supplementary figures and images for: De Novo variants in the KMT2A (MLL) gene causing atypical Wiedemann-Steiner syndrome in two unrelated individuals identified by clinical exome sequencing
Source: BMC Med Genet. 2014 May 1;15:49. doi: 10.1186/1471-2350-15-49 (PMC4072606; doi:10.1186/1471-2350-15-49)

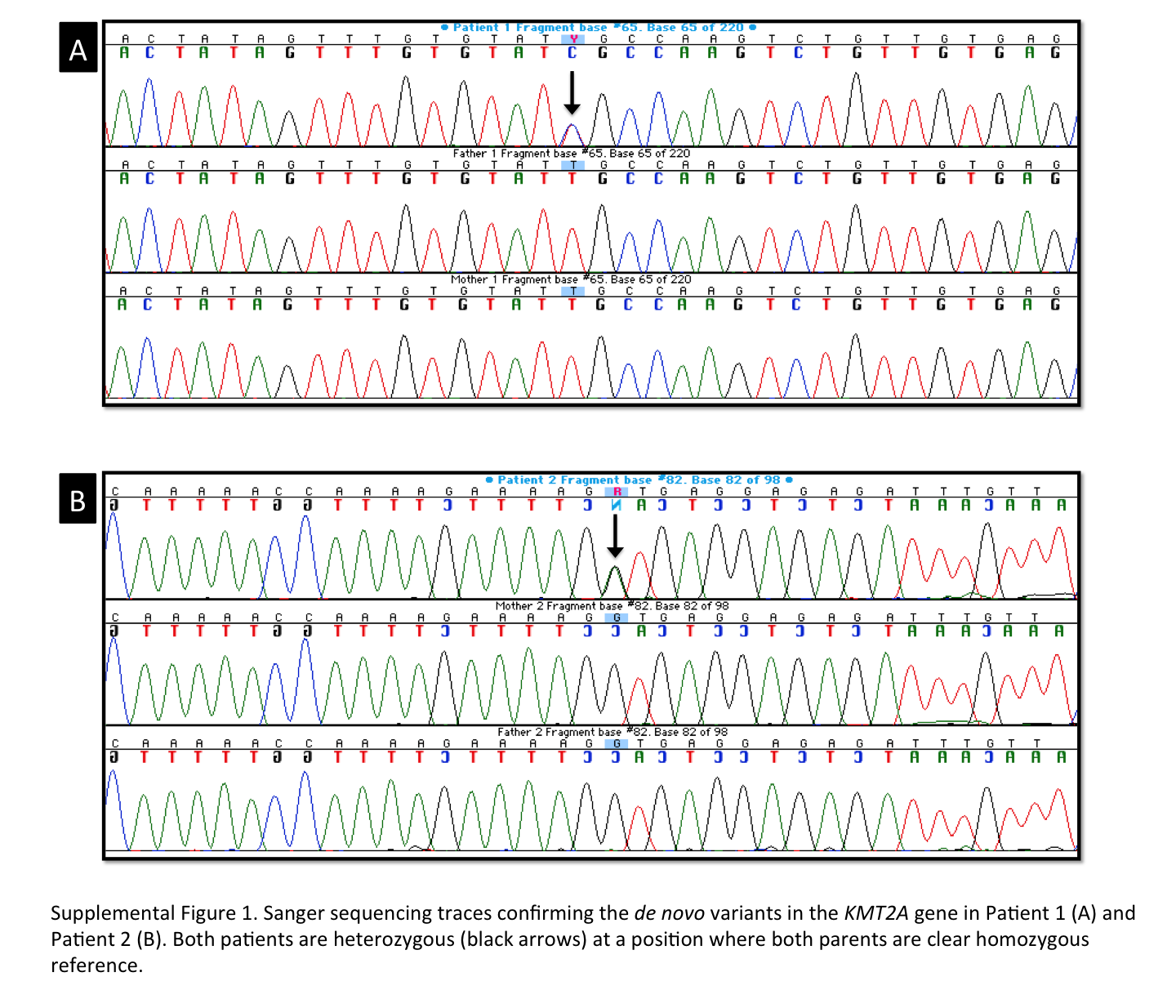

Supplement: Additional file 2 — Sanger sequencing traces confirming de novo variants in the KMT2A gene. [file 1471-2350-15-49-S2.png]
