# Supplementary material for: De Novo variants in the KMT2A (MLL) gene causing atypical Wiedemann-Steiner syndrome in two unrelated individuals identified by clinical exome sequencing
Source: BMC Med Genet. 2014 May 1;15:49. doi: 10.1186/1471-2350-15-49 (PMC4072606; doi:10.1186/1471-2350-15-49)
Supplement: Additional file 3 — Primary gene list for patients 1 and 2. [file 1471-2350-15-49-S3.doc]

NOTE: The gene symbol *MLL* is synonymous with *KMT2A*

Supplemental Materials 2a: Primary gene list for Patient 1

*ABAT, ABCC8, ABCC9, ABCD4, ACAD9, ACADM, ACADS, ACADVL, ACAT1, ACBD6, ACO2, ACOX1, ACOX2, ACP5, ACSF3, ACSL4, ACTA1, ACTB, ACTC1, ACTN2, ACY1, ADAMTS2, ADCK3, ADCYAP1, ADH1A, ADK, ADRA2B, ADSL, AFF2, AFG3L2, AGK, AGL, AGPAT2, AGPS, AGT, AGTR2, AHI1, AIMP1, AKAP13, ALDH18A1, ALDH5A1, ALDOA, ALG1, ALG11, ALG12, ALG3, ALG6, ALG8, ALG9, ALPL, ALPP, ALX1, ALX3, ALX4, AMER1, AMMECR1, AMPD1, ANK1, ANKH, ANKRD1, ANKRD11, ANO5, AP1S1, AP1S2, AP3B1, AP4B1, AP4E1, AP4M1, AP4S1, APAF1, APOB, APOBEC2, APP, APPL1, AR, ARFGEF2, ARHGEF6, ARHGEF9, arhgef9tv2, ARID1A, ARID1B, ARIH1, ARSB, ARSE, ARVCF, ARX, ASCC3, ASCL1, ASPA, ASPM, ASS1, ASXL1, ATCAY, ATIC, ATL1, ATM, ATN1, ATP10A, ATP13A2, ATP1A2, ATP1A3, ATP2A1, ATP5E, ATP6, ATP6AP2, ATP6V0A2, ATP7A, ATP8A2, ATPAF2, ATR, ATRNL1, ATRX, ATXN3, AUTS2, AVIL, AVPR2, B3GALTL, B3GAT3, B4GALT1, BAG3, BARX2, BBS7, BBS9, BCAP31, BCKDK, BCS1L, BDNF, BECN1, BIN1, BLM, BMP4, BNC2, BNIP3, BRAF, BRAFPS2, BRWD3, BSCL2, BUB1B, C10orf11, C10orf2, C11orf46, C12orf57, C12orf65, C5orf42, C6, C9orf86, CA2, CA8, CACNA1A, CACNA1D, CACNA1G, CACNA1S, CACNG2, CALM1, CAMTA1, CANT1, CAPN3, CASK, CASP2, CASP7, CAV3, CBL, CBS, CC2D1A, CC2D2A, CCDC22, CCDC78, CCDC8, CCDC88C, CCS, CDH15, CDIP1, CDK11A, CDK19, CDK5R1, CDKL3, CDKL5, CDON, CENPJ, CEP135, CEP164, CEP290, CEP41, CEP57, CFDP1, CFL2, CFTR, CGA, CGGBP1, CHAT, CHD2, CHD6, CHD7, CHKB, CHL1, CHMP1A, CHMP2B, CHRD, CHRNA1, CHRNA4, CHRNA7, CHRNB1, CHRNE, CHRNG, CHUK, CIC, CLIC2, CLIP2, CLK2, CLN8, CLTCL1, CMIP, CNBP, CNKSR1, CNKSR2, CNTN1, CNTN4, CNTNAP2, COA3, COG4, COG5, COG7, COL11A1, COL11A2, COL18A1, COL1A1, COL2A1, COL5A1, COL6A1, COL6A2, COL6A3, COL7A1, COL9A2, COL9A3, COLEC11, COMP, COMT, COQ4, COQ5, COX1, COX10, COX14, COX15, COX2, COX3, COX7B, CPS1, CPT2, CRADD, CRBN, CREBBP, CRK, CRKL, CRLF1, CRX, CRYAA, CRYAB, CSF1, CSRP3, CTH, CTNND2, CTSA, CUL4B, CUX2, CYB5R3, CYFIP1, CYFIP2, CYP26B1, CYP27A1, CYP27B1, CYTB, D2HGDH, DACT3, DAG1, DARS2, DCAF17, DCX, DDHD2, DDOST, DDR1, DEAF1, DECR1, DES, DGKD, DGUOK, DHCR24, DHCR7, DHTKD1, DIO1, DIP2B, DISC1, DISP1, DLD, DLG1, DLG3, DLGAP2, DLK1, DLL3, DLX1, DLX2, DLX5, DLX6, DMD, DMPK, DNAJB6, DNAJC6, DNM2, DNMT3B, DOCK3, DOCK8, DOK7, DOLK, DPAGT1, DPF1, DPF2, DPF3, DPYD, DPYS, DRD4, DSC2, DSCAM, DSCR3, DSG2, DSP, DST, DUX4, DYM, DYNC1H1, DYRK1A, DYSF, E2F4, EARS2, EBP, ECE1, ECEL1, EDA, EDA2R, EDN1, EDNRA, EEF1B2, EFHC2, EFNB1, EFTUD2, EGR2, EGR3, EHMT1, EIF2C1, EIF2S3, ELK1, ELN, ELOVL4, ELP2, EMX2, ENTPD1, EOMES, EP300, EPAS1, EPB41L1, EPC2, EPHA5, EPHA7, EPHB2, EPHB3, EPHX1, ERCC2, ERCC5, ERCC6, ERCC8, ERLIN2, ESCO2, ETFDH, EVC2, EXOSC3, EXT1, EXT2, EYA1, EYA4, EZH2, F9, FAAH2, FAM126A, FAM58A, FANCA, FAS, FASN, FASTKD2, FBN1, FBN2, FGD1, FGF10, FGF14, FGF8, FGFR1, FGFR2, FGFR3, FGFRL1, FH, FHL1, fhl1tv1, FKBP14, FKRP, FKTN, FLII, FLNA, FLNB, FLNC, FLVCR1, FMN2, FMO3, FMR1, FOLR1, FOS, FOXC1, FOXC2, FOXG1, FOXL2, FOXO1, FOXO3, FOXO4, FOXP1, FOXP2, FOXRED1, FREM1, FRG2, FRMD7, FRMPD4, FRY, FRZB, FTL, FTO, FTSJ1, FXR1, FXR2, G6PD, GABRE, GAL, GALC, GALE, GAMT, GAN, GAS1, GATA3, GATA4, GATA6, GBA, GBE1, GCH1, GCK, GDF5, GDI1, GDI2, GFER, GFM1, GH1, GHRHR, GJA1, GLI2, GLI3, GLIS1, GLO1, GLRA1, GLYCTK, GM2A, GNA11, GNAQ, GNAS, GNE, GNPAT, GNPTAB, GNRHR, GNS, GON4L, GPC3, gpd2c, GPHN, GPI, GPR143, GPSM2, GRIA2, GRIA3, GRIK2, GRIN1, GRIN2A, GRIN2B, GRM1, GSC, GSK3B, GSPT2, GSS, GTF2I, GTF2IRD1, GUSB, GYS1, H6PD, HADHA, HADHB, HAND2, HAPLN1, HAX1, HBA1, HCFC1, HDAC4, HDAC8, HDX, HEPACAM, HEXA, HEXB, HIBCH, HIC1, HIST3H3, HIVEP2, HMBS, HMGA2, HMX1, HMX2, HMX3, HNRNPU, HOXA1, HOXA7, HOXB5, HOXB6, HOXD1, HOXD13, HPD, HPRT1, HPSE2, HRAS, HSD17B10, HSPD1, HSPG2, HUWE1, IDS, IFT140, IGBP1, IGF1, IGF1R, IGFBP1, IGHMBP2, IL11RA, IL1RAPL1, INHBA, INHBB, inpp4ab, INPP5E, INSR, INVS, IQSEC2, IRF6, IRX5, ISCU, ISPD, ITCH, ITGA7, ITPR1, JAG1, JAG2, JAM3, JUP, KAL1, KANK1, KANSL1, KARS, KAT6A, KAT6B, KBTBD13, KCNC3, KCNE1L, KCNJ10, KCNJ11, KCNJ2, KCNK9, KCNQ1, KCNQ2, KCNQ3, KCNT1, KCTD7, KDM5A, KDM5C, KDM6B, KDR, KIAA1033, KIAA1279, KIAA2022, KIF11, KIF1A, KIF21A, KIF3A, KIF5A, KIF7, KIRREL3, KIT, KLF8, KLHL9, KRAS, KY, L1CAM, L2HGDH, LAMA1, LAMA2, LAMB2, LAMP2, LARGE, LBR, LDB2, LDB3, LETM1, LHX3, LHX8, LHX9, LIAS, LIG4, LIMK1, LINS, LMBRD1, LMNA, LPIN1, LPL, LRFN5, LRP4, LRP5, LRRC8A, LTBP3, LTBP4, MAB21L1, MAGED2, MAGEL2, MAGI2, MAGT1, MAMLD1, MAN1B1, MAN2B1, MANBA, MAOA, MAOB, MAP2, MAP2K1, MAP2K2, MAPK14, MAPT, MASP1, MAT1A, MATR3, MBD5, MBTPS2, MCCC1, MCCC2, MCL1, MCPH1, MECP2, mecp2e1, MED12, MED13, MED13L, MED23, MEF2C, MEGF10, MEN1, MEOX1, MEOX2, MEST, MFF, MGAT2, MGAT4C, MID1, MIPOL1, MIR140, MIR17HG, MIR369, MKKS, MKX,* ***MLL****, MLLT3, MLYCD, MMADHC, MMP14, MMP2, MNT, MNX1, MOCS2, MOGS, MPC1, MPPED2, MPZ, MRPS22, MSMO1, MSX1, MSX2, MT-ATP8, MTFMT, MTHFR, MTM1, MTMR1, MTMR14, MTMR2, MTMR9, MTR, MTRR, MT-TE, MT-TG, MT-TH, MT-TI, MT-TK, MT-TL1, MT-TL2, MT-TT, MUSK, MVK, MYBPC3, MYCN, MYF6, MYH14, MYH2, MYH3, MYH6, MYH7, MYH8, MYL2, MYL3, MYL7, MYLK2, MYO15A, MYO5A, MYOT, MYOZ2, MYT1, MYT1L, NAA10, NAGA, NAGLU, NBEA, ND1, ND2, ND3, ND5, ND6, NDN, NDP, NDST1, NDUFA1, NDUFA10, NDUFA12, NDUFA2, NDUFA8, NDUFAF1, NDUFAF3, NDUFB3, NDUFS1, NDUFS3, NDUFS4, NDUFS8, NDUFV2, NEB, neb170, neb171, neb172, NEBL, NEK1, NEXN, NF1, NFE2, NFIA, NFIX, NFKB1, NGEF, NGF, NGLY1, NHEJ1, NHP2, NHS, NIN, NIPBL, NKAIN2, NKX2-1, NKX3-2, NLGN1, NLGN3, NLGN4X, NMNAT1, NOS1, NOTCH1, NOTCH2, NPAS4, NPC2, NPHP1, NPHP3, NR0B1, NR5A1, NRAS, NRXN1, nrxn1b, NSD1, NSDHL, NSMF, NSUN2, NTM, NTRK2, NUBPL, NUFIP1, NUFIP2, NXF5, OCA2, OCLN, OCRL, OFD1, OMG, OPA1, OPHN1, OSR2, OTX2, P2RY8, PABPN1, PACS1, PAFAH1B1, PAFAH1B3, PAH, PAK2, PAK3, PARP1, PARVB, PAX2, PAX3, PAX6, PAX9, PCCB, PCDH11X, PCDH18, PCDH19, PCNT, PDE4D, PDGFC, PDHA1, PDHX, PDSS1, PECR, PEPD, PEX10, PEX11B, PEX12, PEX13, PEX14, PEX19, PEX3, PEX5, PEX6, PEX7, PGK1, PGRMC1, PHEX, PHF21A, PHF6, PHF8, PHKA1, PHKG2, PHOX2B, PIGA, PIGL, PIGN, PIGO, PIGV, PITX1, PITX2, PKD1L2, PKP2, PLA2G6, PLEKHM1, PLN, PLOD1, PLOD3, PLP1, PLP2, PMM2, PMP22, PNKP, PNP, PNPLA2, PNPO, PNPT1, POLG, POLG2, POLR3A, POLR3B, POMGNT1, POMT1, POMT2, POR, PORCN, POU1F1, POU3F1, POU3F2, POU3F4, PPID, PPOX, PPP2R2C, PQBP1, PRDM16, PREPL, PRICKLE1, PRICKLE2, PRKAG2, PRKAR1A, PRKCG, PRKRA, PRL, PRMT10, PROP1, PRPS1, PRRX1, PRSS12, PSAP, PSEN1, PSEN2, PSMG1, PTCH1, PTCHD1, PTEN, PTH, PTH1R, PTHLH, PTPLA, PTPN11, PTPRF, PTPRS, PUS1, PVRL1, PYCR1, QDPR, QKI, RAB18, RAB39B, RAB3GAP2, RAB40AL, RABL2B, RAD21, RAI1, RALGAPA1, RALGDS, RAPSN, RARS2, RASGEF1B, RBBP8, RBFOX1, rbfox1i4, RBM20, RDH10, RELN, REPS2, REST, RGS7, RHD, RMND1, RMRP, RNF135, RNU4ATAC, ROGDI, ROR2, RP2, RPGR, RPGRIP1L, RPS6KA3, RPS6KA6, RRM2B, RTTN, RUNX1, RUNX1T1, RUNX2, RYR1, RYR2, SACS, SALL1, SALL4, SAMHD1, SAT1, SATB2, SC5DL, SCN1A, SCN2A, SCN4A, SCN5A, SCN8A, SCO2, SDCCAG8, SDHA, SDHB, SDHC, SEC23A, SEL1L, SEPN1, SEPSECS, SEPT9, SERAC1, SETBP1, SETD5, SFN, SGCA, SGCB, SGCD, SGCE, SGSH, SH2B1, SH3PXD2B, SHANK2, SHANK3, SHH, SHOC2, SHOX, SHROOM4, SIL1, SIM2, SIX3, SKI, SLC16A2, SLC17A5, SLC1A1, SLC1A3, SLC20A2, SLC22A5, SLC25A15, SLC25A3, SLC25A4, SLC29A3, SLC2A1, SLC2A2, SLC31A1, SLC33A1, SLC35C1, SLC35D1, SLC3A1, SLC46A1, SLC4A10, SLC4A4, SLC52A3, SLC5A2, SLC6A1, SLC6A3, SLC6A8, SLC7A7, SLC9A6, slc9a6tv1, SLC9A9, SLCO1B1, SLX4, SMAD4, SMARCA2, SMARCA4, SMARCB1, SMC1A, SMC3, SMS, SNIP1, SNORD116-1, SNX3, SOBP, SOD2, SOS1, SOSTDC1, SOX10, SOX14, SOX2, SOX3, SOX5, SOX8, SOX9, SP7, SP8, SPG20, SPINT2, SPR, SPRED1, SPTAN1, SPTLC1, SRD5A3, SRGAP2, SRGAP2C, SRGAP3, SRPX2, ST2, ST3GAL3, ST5, STAG1, STIL, STIM1, STK11, STRA6, STS, STXBP1, SUCLG1, SUFU, SUMO1, SUPT3H, SURF1, SUZ12, SYNCRIP, SYNE1, SYNE2, SYNGAP1, SYP, SYT14, TAF2, TAZ, TBC1D24, TBCE, TBX1, TBX15, TBX2, TBX22, TBX3, TCAP, TCF4, TCN2, TCOF1, TDGF1, TECPR2, TECR, TERT, TFAM, TFAP2A, TFAP2B, TFAP2C, TGFB2, TGFB3, TGFBR1, TGFBR2, TGIF1, TH, THRB, TIMM8A, TINF2, TK2, TMCO1, TMEM135, TMEM165, TMEM185A, TMEM216, TMEM237, TMEM43, TMEM67, TMEM70, TMLHE, TMPO, TNFSF11, TNNC1, TNNI3, TNNT1, TNNT2, TNR, TNS3, TOR1A, TP63, TPI1, TPK1, TPM1, TPM2, TPM3, TPO, TRAF6, TRAPPC9, TRIM28, TRIM32, TRMT1, TRMU, TRNA, TRNC, TRND, TRNE, TRNF, TRNG, TRNH, TRNI, TRNK, TRNL1, TRNL2, TRNM, TRNN, TRNP, TRNQ, TRNR, TRNS1, TRNS2, TRNT, TRNV, TRNW, TRNY, TRPC5, TRPM3, TRPS1, TSC1, TSC2, TSFM, TSHR, TSHZ1, TSPAN7, TTC37, TTI2, TTN, TUB, TUBA1A, TUBA8, TUBB2B, TUBB3, TUSC3, TWIST1, TWIST2, TXNRD2, UBA1, UBB, UBE2A, UBE3A, UBR1, UBR7, UCP3, UFD1L, UPB1, UPF3A, UPF3B, UQCRC1, UROC1, VCL, VCP, VCX3A, VEGFA, VLDLR, VMA21, VPS13A, VPS13B, VPS33B, VRK1, VSIG4, VSX1, WDPCP, WDR13, WDR35, WDR45L, WDR62, WDR65, WDR81, WHSC1, WNT3, WNT5A, WTIP, XIST, XK, XPNPEP3, YARS2, YPEL1, YWHAE, YY1, ZBTB16, ZBTB24, ZBTB40, ZC3H14, ZCCHC12, ZCCHC8, ZDHHC15, ZDHHC9, ZEB2, ZFHX4, ZFP57, ZFP90, ZFYVE1, ZFYVE26, ZIC2, ZIC3, ZMYM3, ZMYM4, ZMYM6, ZNF146, ZNF238, ZNF385B, ZNF41, ZNF526, ZNF630, ZNF674, ZNF711, ZNF81*

Supplemental Materials 2b: Primary gene list for patient 2

*A1CF, AAAS, ABAT, ABCB11, ABCB6, ABCC8, ABCC9, ABCD4, ABCG5, ABHD5, ACACA, ACAD8, ACAD9, ACADM, ACADS, ACADSB, ACADVL, ACAT2, ACO2, ACOX1, ACP2, ACP5, ACSF3, ACTA1, ACTA2, ACTB, ACTRT1, ACY1, ADAMTS18, ADAMTS2, ADAMTSL2, ADAR, ADCK3, ADK, ADSL, AFF2, AGA, AGK, AGL, AGPS, AGT, AGTR1, AGXT2L2, AHCY, AHI1, AIC, AIFM1, AIMP1, AIRE, AKAP13, AKT1, AKT3, ALAD, ALDH18A1, ALDH1A3, ALDH5A1, ALDH7A1, ALDOB, ALG1, ALG11, ALG12, ALG3, ALG6, ALG8, ALG9, ALMS1, ALPL, ALX1, ALX3, AMACR, AMER1, AMPD1, AMT, ANK1, ANKRD11, ANOP1, ANTXR1, AP1S1, AP1S2, AP3B1, AP4B1, AP4E1, AP4M1, AP4S1, APEX2, APOA1, APOC2, APOL2, APOL4, APPL1, AR, ARFGEF2, ARFRP1, ARG1, ARHGAP31, ARHGAP6, ARHGEF9, ARID1A, ARID1B, ARID5B, ARL6, ARL6IP1, ARSA, ARSB, ARSE, ARX, ASAH1, ASIP, ASL, ASPA, ASPH, ASS1, ASXL1, ATCAY, ATF4, ATIC, ATL1, ATM, ATOX1, ATP10A, ATP1A2, ATP1A3, ATP2B3, ATP6V0A2, ATP7A, ATP7B, ATP8B1, ATPAF2, ATR, ATRNL1, ATRX, ATXN1, ATXN2, ATXN3, ATXN7, AUH, AUTS1, AUTS2, AVP, B3GALTL, B3GAT3, B4GALT1, B4GALT7, B9D1, BACE1, BANF1, BARD1, BBS1, BBS10, BBS12, BBS2, BBS4, BBS5, BBS7, BBS9, BCAR1, BCKDHA, BCKDHB, BCKDK, BCOR, BCS1L, BECN1, BEST1, BIN1, BIRC6, BLM, BMP1, BMP2, BMP4, BMP6, BMPER, BMPR1A, BMPR1B, BPIFA2, BRAF, BRCA1, BRCA2, BRCC3, BRIP1, BRWD3, BSND, BTD, BUB1B, C10orf2, C12orf57, C12orf65, C21orf33, C5orf42, CA2, CACNA1A, CACNA1C, CACNA1S, CACNA2D2, CAMTA1, CANT1, CASK, CASP3, CASR, CAV3, CBL, CBS, CC2D1A, CC2D2A, CCBE1, CCDC28B, CCDC78, CCDC8, CCDC88C, CCND1, CCNE1, CCS, CD3D, CD3E, CD40LG, CD9, CD96, CDAN1, CDC42, CDC6, CDH15, CDH3, CDK4, CDKL5, CDKN1C, CDON, CDT1, CECR, CELSR3, CENPJ, CEP135, CEP164, CEP290, CEP41, CEP57, CFC1, CFH, CFL1, CFL2, CFLAR, CGF1, CHAT, CHD2, CHD7, CHI3L1, CHKB, CHL1, CHMP1A, CHRM3, CHRNA1, CHRNA7, CHRNB1, CHRND, CHRNE, CHRNG, CHST14, CHST3, CHSY1, CIB2, CLAM, CLCF1, CLCN4, CLCN5, CLCNKA, CLCNKB, CLIC2, CLIP2, CLN3, CLN8, CMC4, CMIP, CNGB3, CNTN1, CNTN4, CNTNAP2, COA3, COA5, COG1, COG4, COG5, COG7, COG8, COL11A1, COL11A2, COL18A1, COL1A1, COL1A2, COL2A1, COL3A1, COL4A1, COL4A2, COL5A1, COL5A2, COL6A1, COL6A2, COL6A3, COL7A1, COL9A2, COLEC11, COLQ, COMP, COMT, COPG2, COQ2, COQ4, COQ6, COQ9, COX10, COX14, COX15, COX20, COX4I2, COX6B1, COX7B, CPA4, CPT1A, CPT2, CRB1, CREB1, CREBBP, CRELD1, CRH, CRHR1, CRK, CRLF1, CRTAP, CRX, CRYAA, CRYAB, CRYBA4, CRYBB2, CSF2RA, CTC1, CTDP1, CTLA4, CTNNB1, CTNND2, CTNS, CTSA, CTSK, CUL4B, CUL7, CYB5R3, CYP11B2, CYP1B1, CYP21A2, CYP24A1, CYP27B1, CYP2R1, D2HGDH, DAB2IP, DACH1, DAG1, DAO, DAOA, DARS2, DBH, DBT, DCR, DCX, DDC, DDOST, DDR2, DDX11, DECR1, DGCR2, DGKD, DGUOK, DHCR24, DHCR7, DHFR, DHODH, DHTKD1, DIH1, DIO2, DIP2B, DIS3L2, DISC1, DISC2, DISP1, DKC1, DLAT, DLD, DLG1, DLK1, DLL3, DLX5, DMD, DMPK, DNAH11, DNAJB2, DNM1L, DNM2, DNMT3B, DOCK6, DOK7, DOLK, DPAGT1, DPM1, DPM2, DPYD, DPYS, DRD2, DRD3, DSG4, DST, DTNA, DTNB, DTNBP1, DURS1, DWS, DYM, DYNC1H1, DYRK1A, DYSF, EARS2, EBP, ECEL1, EDN3, EDNRB, EEC1, EFEMP2, EFNB1, EFTUD2, EGF, EGR2, EHMT1, EHMT2, EIF2AK3, EIF2B1, EIF2B2, EIF2B3, EIF2B4, EIF2B5, ELN, ELOVL4, EMD, EMG1, EMX2, ENTPD2, EOMES, EP300, EPB41L1, EPCAM, EPG5, EPHA7, EPHX1, ERBB3, ERCC1, ERCC2, ERCC3, ERCC5, ERCC6, ERCC8, ESCO2, ESX1, ETFA, ETFB, ETFDH, ETHE1, EXOSC3, EXT1, EYA1, EZH2, F5, F8, F8A1, FAH, FAM107A, FAM123B, FAM126A, FAM20A, FAM20C, FAM3C, FAM58A, FAN1, FANCA, FANCB, FANCD2, FANCE, FANCG, FANCL, FARS2, FASTKD2, FBLN1, FBLN5, FBN1, FBN2, FBP1, FBXW4, FERMT1, FGD1, FGF10, FGF23, FGF3, FGFR1, FGFR2, FGFR3, FGFRL1, FH, FHL1, FIG4, FKBP14, FKRP, FKTN, FLII, FLNA, FLNB, FLVCR2, FMN1, FMR1, FOXC1, FOXC2, FOXE3, FOXF1, FOXG1, FOXL1, FOXL2, FOXO3, FOXP1, FOXP2, FOXP3, FOXRED1, FRAS1, FREM1, FREM2, FTCD, FTL, FTO, FUCA1, FUT8, FZD4, G6PC, G6PC3, GAA, GAL, GALC, GALE, GALNTL5, GALT, GAMT, GATA1, GATA2, GATA3, GATA4, GATA6, GATAD2B, GATM, GBA, GBE1, GCDH, GCH1, GCK, GCSH, GDAP1, GDF1, GDF3, GDF5, GDF6, GFAP, GFER, GFM1, GFRA2, GH1, GHR, GHRHR, GJA1, GJA8, GJB2, GJB4, GJC2, GK, GLB1, GLDC, GLE1, GLI2, GLI3, GLIS3, GLUL, GLYCTK, GM2A, GNAI2, GNAI3, GNAS, GNE, GNPAT, GNPTAB, GNPTG, GNS, GOSR2, GPC3, GPC6, GPD1, GPHN, GPR143, GPR56, GPSM2, GPX4, GRB10, GRIA3, GRIK2, GRIK5, GRIN1, GRIN2A, GRIN2B, GRIP1, GRM1, GTDC2, GTF2H5, GTF2IRD1, GTS, GUCY2D, GUSB, H19, HADH, HADHA, HADHB, HAP1, HAX1, HBB, HBD, HCAR1, HCCS, HDAC4, HDAC8, HELLS, HEPACAM, HERC2, HES7, HESX1, HEXA, HFE, HGF, HIBCH, HIC1, HIF1A, HIVEP2, HIVEP3, HLA-DQA1, HLA-DQB1, HLCS, HMBS, HMGA2, HMOX1, HMX1, HMX2, HMX3, HOXA1, HOXA11, HOXB1, HOXB6, HOXD1, HOXD13, HPD, HPE1, HPGD, HPRT1, HR, HRAS, HRSP12, HSD11B2, HSD17B10, HSD17B4, HSPD1, HSPG2, HTR2A, HUWE1, HYLS1, HYMAI, IBGC1, ICK, IDH2, IDUA, IER3IP1, IFNG, IFT122, IFT43, IGBP1, IGF1, IGF1R, IGF2, IGFBP1, IGFBP5, IGFBP7, IGHMBP2, IGSF1, IHH, IKBKAP, IKBKG, IL10, IL11RA, IL1RAPL1, IL6, IL7R, IL9R, IMPAD1, IMPDH1, INPP5E, INPPL1, INS, INSR, INVS, IRF6, IRX5, ISL1, ISPD, ITCH, ITGA7, ITPR1, IVD, IYD, JAG1, JAG2, JAM3, JBS, KANK1, KANSL1, KANSL3, KARS, KAT6B, KCNC3, KCNJ1, KCNJ10, KCNJ11, KCNJ13, KCNJ2, KCNK9, KCNQ1, KCNQ1OT1, KCNQ2, KCNT1, KDM5C, KDM6A, KERA, KIAA1279, KIF11, KIF1A, KIF22, KIF26A, KIF3B, KIF5B, KIF7, KIRREL3, KIT, KMT2A, KMT2B, KMT2D, KRAS, KRT14, KRT18, KRT5, KRT8, L1CAM, LAMA2, LAMA3, LAMB1, LAMB2, LAMB3, LAMC1, LAMC2, LARGE, LARP7, LATS1, LBR, LEF1, LEP, LEPREL1, LGR4, LHX3, LHX4, LIAS, LIFR, LIG1, LIG4, LINC00299, LMBR1, LMBRD1, LMNA, LMO7, LPIN1, LPL, LRBA, LRFN5, LRP2, LRP4, LRP5, LRP6, LRPPRC, LRRC7, LTBP2, LTBP3, LTBP4, LTC4S, LYST, MAATS1, MAB21L1, MAF, MAFB, MAGI2, MALT1, MAMLD1, MAN2B1, MANBA, MAOA, MAOB, MAP2, MAP2K1, MAP2K2, MAPK10, MAPT, MASP1, MAT1A, MBD5, MBOAT1, MBS1, MBTPS2, MCCC1, MCCC2, MCM4, MCOLN1, MCPH1, MDC1, MECP2, MED1, MED12, MEF2C, MEG3, MEGF10, MEGF8, MEN1, MEOX1, MEST, MFF, MFRP, MGAT2, MID1, MIPOL1, MIR137, MIR17, MIR17HG, MIR182, MIR200C, MIR204, MITF, MKKS, MKS1,* ***MLL****, MLLT3, MLYCD, MMAA, MMAB, MMACHC, MMADHC, MMP1, MMP14, MMP2, MMVP1, MNX1, MOCS1, MOCS2, MOGS, MPC1, MPDU1, MPI, MPLKIP, MPV17, MPZ, MRPS16, MRPS22, MRT23, MRT24, MRT4, MRT9, MRX14, MRXS11, MRXS5, MSMB, MSMO1, MSX2, MTCP1, MTFMT, MTHFR, MTM1, MTMR14, MTO1, MTR, MTRR, MUSK, MUT, MVK, MYCN, MYH3, MYH8, MYMY1, MYO5A, MYO6, MYP2, NAA10, NAGA, NAGLU, NAGS, NAMSD, NAT8L, NBAS, NBN, NCAM1, NCOA2, NCOA6, NDE1, NDN, NDP, NDUFA1, NDUFA10, NDUFA11, NDUFA12, NDUFA13, NDUFA2, NDUFA8, NDUFA9, NDUFAF1, NDUFAF2, NDUFAF3, NDUFAF4, NDUFAF5, NDUFAF6, NDUFB3, NDUFS1, NDUFS2, NDUFS3, NDUFS4, NDUFS6, NDUFS7, NDUFS8, NDUFV1, NDUFV2, NEB, NEBL, NEFL, NEK1, NEU1, NF1, NFIA, NFIX, NFKBIA, NGLY1, NHEJ1, NHP2, NHS, NIN, NIPBL, NKAIN2, NKX2-1, NKX2-5, NKX2-6, NLGN3, NLGN4X, NLRP1, NLRP2, NLRP3, NLRP7, NMNAT1, NNO1, NOD2, NODAL, NOG, NOS1, NOS3, NOTCH2, NPAS4, NPC1, NPC2, NPHP1, NPHP3, NPHP4, NPHS1, NQO1, NR0B1, NR2C2, NR2F1, NR5A1, NRAS, NRG2, NRXN1, NSD1, NSDHL, NSUN2, NTRK1, NTRK2, NUBPL, NUP133, OCA2, OCLN, OCRL, OFD1, OGDH, OPHN1, ORAI1, ORC1, ORC4, ORC6, OTC, OTX2, OXCT1, PAFAH1B1, PAH, PAK3, PALB2, PARL, PAX2, PAX3, PAX6, PBCRA1, PC, PCBD1, PCCA, PCCB, PCDH19, PCK1, PCK2, PCMT1, PCNT, PDCD6IP, PDE4D, PDE6B, PDGFRA, PDHA1, PDHB, PDP1, PDR, PDSS2, PDX1, PDZD7, PEE1, PEPD, PEX1, PEX10, PEX11B, PEX12, PEX13, PEX14, PEX16, PEX19, PEX2, PEX26, PEX3, PEX5, PEX6, PEX7, PGAP1, PGAP2, PGK1, PGM1, PHEX, PHF21A, PHF6, PHF8, PHGDH, PHKA2, PHKB, PHKG2, PHOX2B, PIAS3, PIGA, PIGL, PIGN, PIGO, PIGV, PIK3CA, PIK3R2, PIKFYVE, PITX1, PITX2, PITX3, PKD1, PKHD1, PKLR, PLA2G6, PLAGL1, PLCB1, PLCB4, PLEC, PLG, PLOD1, PLOD3, PLP1, PMM2, PMP22, PMPCA, PNKP, PNP, PNPLA6, PNPO, PNPT1, POC1A, POLE, POLG, POLG2, POLH, POLR3A, POLR3B, POMC, POMGNT1, POMT1, POMT2, POR, PORCN, POU1F1, POU3F1, POU3F2, POU3F4, PPARG, PPOX, PPP1R12A, PPT1, PQBP1, PRDM16, PRDM5, PREPL, PRF1, PRICKLE2, PRKACA, PRKAG2, PRKAR1A, PRKG2, PRLR, PROC, PRODH, PROP1, PROS1, PRPS1, PRRT2, PRRX1, PRS, PRSS56, PRX, PSAP, PSG1, PSMB8, PSMG2, PSPH, PSPN, PTCH1, PTCHD1, PTEN, PTF1A, PTH1R, PTPN11, PTPN14, PTPRC, PTPRF, PTPRS, PTRF, PTS, PUS1, PVRL1, PVRL4, PYCR1, PYGL, PYHIN1, QDPR, QKI, RAB18, RAB23, RAB33B, RAB39B, RAB3GAP1, RAB3GAP2, RAB40AL, RAD21, RAD50, RAG1, RAG2, RAI1, RALGAPA1, RANBP2, RAPSN, RARS2, RAX, RBBP8, RBFOX1, RBM10, RBM8A, RBPJ, RC3H1, RCAN1, RCD1, RECQL4, REG1A, RELN, REN, RET, RFT1, RFX6, RIEG2, RIN2, RIPK4, RMND1, RNF135, RNF168, RNF8, RNU4ATAC, ROCK2, ROGDI, ROR2, RPGRIP1L, RPL5, RPS17, RPS19, RPS6KA3, RRM2B, RS1, RSS, RTEL1, RTN4R, RUNX1, RUNX2, RUNX3, RYK, RYR1, SALL1, SALL4, SAMHD1, SAR1B, SARDH, SARS2, SAT1, SATB2, SBDS, SC5D, SCAR2, SCAR6, SCARF2, SCAX1, SCN1A, SCN1B, SCN2A, SCN4A, SCN8A, SCN9A, SCO2, SCZD1, SCZD2, SCZD3, SCZD5, SCZD6, SCZD7, SCZD8, SDHA, SDHAF1, SDHB, SEC23A, SECISBP2, SEMA3E, SEPN1, SEPSECS, SEPT9, SERAC1, SERPINH1, SETBP1, SF3B4, SGCE, SGK1, SGSH, SH2B1, SH2D1A, SH3PXD2B, SHANK2, SHANK3, SHFM1, SHFM2, SHFM5, SHH, SHOX, SHROOM4, SIAH1, SIL1, SIX3, SIX6, SKI, SKIV2L, SLC12A1, SLC12A6, SLC13A1, SLC16A2, SLC17A5, SLC17A8, SLC19A2, SLC20A2, SLC22A5, SLC25A1, SLC25A12, SLC25A13, SLC25A15, SLC25A19, SLC25A22, SLC25A3, SLC26A2, SLC26A3, SLC29A3, SLC2A1, SLC2A10, SLC30A5, SLC33A1, SLC34A3, SLC35A2, SLC35C1, SLC36A2, SLC39A13, SLC39A4, SLC3A1, SLC46A1, SLC4A1, SLC4A4, SLC4A5, SLC52A2, SLC52A3, SLC5A5, SLC6A19, SLC6A20, SLC6A3, SLC6A8, SLC7A7, SLC7A9, SLC9A6, SLCO2A1, SLITRK1, SLX4, SMAD4, SMAD7, SMARCA2, SMARCA4, SMARCAL1, SMARCB1, SMC1A, SMC3, SMN1, SMOC1, SMPD1, SMS, SNAI2, SNAP29, SNCA, SNIP1, SNORD116-1, SNRPN, SNX27, SNX3, SOLH, SOS1, SOST, SOX10, SOX17, SOX2, SOX3, SOX5, SOX9, SP7, SPG16, SPG20, SPG21, SPG9, SPINK5, SPINT2, SPR, SPRED1, SPTAN1, SPTLC1, SRC, SRCAP, SRD5A3, SRGAP2, SRGAP3, SRRT, ST3GAL3, ST3GAL5, ST5, STAMBP, STAT5B, STEAP3, STIM1, STK11, STOX1, STRA6, STRADA, STS, STXBP1, SUCLA2, SUCLG1, SUFU, SUMF1, SUOX, SURF1, SUZ12, SYN2, SYNE1, SYNGAP1, SYT14, TAF1, TAZ, TBCE, TBX1, TBX15, TBX2, TBX22, TBX4, TBX5, TCF4, TCF7L2, TCIRG1, TCN2, TCOF1, TCTN2, TCTN3, TDGF1, TECPR2, TECR, TECTA, TENM1, TENM3, TERT, TF, TFAP2A, TFAP2B, TFAP2C, TGFB1, TGFB2, TGFBR1, TGFBR2, TGIF1, TH, THRA, THRB, TINF2, TJP2, TK2, TKT, TMCO1, TMEM114, TMEM165, TMEM185A, TMEM216, TMEM231, TMEM237, TMEM67, TMEM70, TNFAIP3, TNFSF11, TNNI2, TNNT1, TNNT3, TNS3, TNXB, TOR1A, TP53, TP63, TPH2, TPI1, TPK1, TPM2, TPM3, TRAPPC9, TREX1, TRIM32, TRIM37, TRMU, TRPM1, TRPS1, TRPV3, TRPV4, TSC1, TSC2, TSEN54, TSFM, TSHB, TSHZ1, TSIX, TSPAN12, TSPYL1, TTC37, TTC8, TTN, TUBA1A, TUBA8, TUBB2B, TUBB3, TUBGCP6, TUFM, TWIST1, UBA1, UBB, UBE2A, UBE3A, UBE3B, UBR1, UCHL3, UFD1L, UMPS, UPB1, UPF3B, UPK3A, UQCRQ, USB1, USH2A, VAX1, VCAN, VDAC1, VDR, VHL, VIPAS39, VLDLR, VMA21, VPS13B, VPS33B, VRK1, VRNI, VSX2, WBS2, WDPCP, WDR19, WDR35, WDR45, WDR62, WFS1, WHSC1, WNK1, WNT1, WNT10B, WNT3, WNT5A, WNT7A, WRN, WS2B, XBP1, XBP1P1, XIST, XPA, YARS2, YWHAE, ZBTB16, ZBTB20, ZBTB24, ZDHHC15, ZDHHC9, ZEB2, ZFP57, ZFP90, ZIC2, ZMPSTE24, ZNF141, ZNF335, ZNF41, ZNF469, ZNF592, ZNF81*
